# Supplementary material for: ZiPo: A Deep Neural Network to De-Noise Single-Cell RNA Sequencing Data
Source: IEEE Trans Comput Biol Bioinform. Author manuscript; Available in PMC 2026 Feb 17. (PMC12910556; doi:10.1109/TCBBIO.2025.3572783)
Supplement: tcbbio-3572783-mm [file NIHMS2129199-supplement-tcbbio-3572783-mm.zip › tcbbio-3572783-mm/code/utilities/OncinfoUt_pack/vignettes/OncinfoUt.html]

OncinfoUt: Facilitating experiments for computational biology


# OncinfoUt: Facilitating experiments for computational biology

Mohsen Tabar and Habil Zare

#### January 17, 2025

# Contents

- 1 Introduction
- 2 The general idea
- 3 Project structure
- 4 Some Oncinfo conventions
  - 4.1 File and path variables
- 5 How to use it in action
  - 5.1 Basic usage
    - 5.1.1 How does the experiment config look like?
    - 5.1.2 How does the task config look like?
  - 5.2 Experiments based on each other
  - 5.3 Common parameters
  - 5.4 Run a task on a grid of parameters
  - 5.5 Using glue to generalize your scripts
  - 5.6 Using a SLURM cluster
  - 5.7 Some notes

# 1 Introduction

We’ve developed *OncinfoUt* package to facilitate reusing pipelines in computational biology
and also easily configure them to have reproducible results without concerns and much effort.
We are using some kind of framework to run the experiments and keep their results.
We also heavily use *YAML* files as configuration of the pipelines.
We encourage you to have a basic knowledge of *YAML* format before continuing.

# 2 The general idea

For any pipeline that you have in mind, first split it to some *task*s.
We call a bunch of these tasks that would be run sequentially to produce the desired results an *experiment*.
To keep the order of the experiments that we may run for any particular pipeline,
we use this pattern for their names: “YYYY-MM-DD\_any\_meaningful\_name\_describing\_the\_purpose”.

The idea is that we have some configurations which are pipeline-related.
For example, list of available tasks to build the pipeline,
or anything that the tasks need outside their codes that are not experiment-specific.
We will give you some examples to better understand this concept.
Anyway, we will keep such settings in a file naming *application.yaml*.
Any experiment has definitely its own settings.
Any parameter, the data to be used as the input, and so on.
We keep those settings in a file naming *{{experiment}}.yaml*.
Therefore, you will have one *application.yaml* file and many experiment yaml files.

At the start of the code of any task, we load these yaml files and merge them
and then return an R list which are the final parameters of that specific task.
Your task should only rely on this list and must reproduce exactly the same results
when called again with the same list. This is really easy to achieve:
Your task code should not use any parameter outside of this list.
Think of your task as a function that has only one big list as the input parameter, and nothing else.

# 3 Project structure

On top of everything, we have a ‘proj’ folder.
Usually it resides on the home folder “~”, but for any reason it may be somewhere else.
For example when we are working on a cluster
and usually the home folder is not enough to keep all the data and results for us.

This folder may contain many projects that we are working on.
In the Oncinfo lab, every project has its own repository which can be cloned.
We specify the project with their names, e.g. ‘alzheimer’, ‘dementia’, etc.

Inside any project, we have three major folders: ‘data’, ‘result’, and ‘code’.
Any data that we may download will be in the data folder.
All the results that we would generate would be in the result folder.
The code folder can have many applications (or subproject let say) related to the main project.
You can structure your applications there.
We usually put the name of the main developer on top,
but any other appropriate structuring is encouraged.

```
proj
└───${proj.name}
│   └───data
│   │   │   ...
│   │
│   └───result
│   │   │   ...
│   │
│   └───code   ## It may have nested structure inside, instead of a flat one.
│   │   │
│   │   └───${application1}
│   │   │   │ ...
│   │   │
│   │   └───${application2}
│   │   │   │ ...
│   │   │
│   │   └───...
```

Any application has the following internal structure:

```
│
│   runall.R
│   libraries.R
│   settings.R
│   application.yaml
│
└───script  ## It may have nested structure inside, instead of a flat one.
│   │   ${task1}.R
│   │   ${task2}.R
│   │   ...
│
└───experiments
│   │   ${date1}_${name1}.yaml
│   │   ${date2}_${name2}.yaml
│   │   ...
│
```

The ‘runall.R’ script is your application interactive entry point.
Whoever wants to run your app and reproduce the results would start from sourcing this script.
In ‘libraries.R’ we would call `library()` on any package that we would need in our app.
In ‘application.yaml’ and ‘settings.R’, we do application-specific configurations.
We will describe it in more details later.
The ‘script’ folder contains split tasks for your pipeline. It only contains scripts, not just R scripts though.
You may have bash scripts for example if needed.
The ‘experiments’ folder contains yaml files which lead to the desired results for each experiment.

# 4 Some Oncinfo conventions

These conventions are highly recommended in the Oncinfo lab.
Outside the lab, feel free to use them or use your own conventions.

## 4.1 File and path variables

We use these conventions for the naming of variables related to file and paths.
These are suffixes for the variables names corresponding to files or paths.
Anything about files might be with or without extension.

- “Path”: Absolute path to a directory. Example: `projPath <- "~/proj/alzheimer"`
- “Folder”: Usually just a name, without slashes. But works if it contains slashes.
  Examples: `experimentFolder <- "2024-01-01_salmon_my_data"` or `experimentFolder <- "salmon/2024-01-01_my_data"`
- “MidPath”: Anything that is not absolute (Not starting with slash or Tilda).
  Examples: `resultMidPath <- "limma"` or `resultMidPath <- "genesis/association"`
- “File”: Absolute path to a file. Example: `makeFile <- "~/proj/alzheimer/code/utilities/makeOncinfoUt.R"`
- “RelativeFile”: Relative path to a file. Example: `makeRelativeFile <- "code/utilities/makeOncinfoUt.R"`
- “FileName”: Just a name of a file. Example: `makeFileName <- "makeOncinfoUt.R"`

# 5 How to use it in action

We illustrate the application of OncinfoUt package
by developing an example pipeline to do differentially expressed (DE) analysis.

The examples that we are showing here are available in the OncinfoUt package files.
To know where you have installed the package OncinfoUt,
you may use the function `find.package("OncinfoUt")` in your R session.
So, the files used for the basic example can be found in the following folder.
We recommend to have these files open when reading the rest of this vignette.

```
dir(file.path(find.package("OncinfoUt"), "extdata/examples/proj/OncinfoUtExamples_DE/code/1_basic"))
```

```
## [1] "application.yaml" "experiments"      "libraries.R"      "runall.R"        
## [5] "script"           "settings.R"
```

For convenience, we also use this helper function in the following examples:

```
getExampleResource <- function(...) {
    system.file("extdata/examples/proj/OncinfoUtExamples_DE/code", ..., package="OncinfoUt")
}
```

## 5.1 Basic usage

Let’s investigate our main script, *runall.R*:

```
## Load all required libraries throughout all the tasks.
source("libraries.R")

## Interactively choose one of the experiments in your experiment folder.
experiment <- setExperiment()

## Load settings in a list named 'config'.
source("settings.R")

## Use the 'tasks' field in the experiment yaml file.
tasks <- config$tasks
## Optional. Allow the user to interactively choose a subset of tasks.
tasks <- chooseFrom(what="task", allOptions=tasks, doAllowMultiple=TRUE)

## Now loop over all tasks
for (task in tasks[tasks!="none"]) {
    ## Load the "merged" task parameters.
    taskParams <- getTaskParams(config=config, task=task)
    ## Create the output directory of this task
    dir.create(taskParams[['taskPath']], recursive=TRUE, showWarnings=FALSE)
    ## Run the task's code.
    source(file.path("script", paste0(task, ".R")))
}
```

The first part is sourcing the ‘libraries.R’ file. Look at the content of this file:

```
library("OncinfoUt")
called <- caLibraries(packages=c("logger", "tictoc",
                                 "Pigengene", "limma", "pheatmap", "SummarizedExperiment",
                                 "org.Hs.eg.db"))
```

As you can see, we first `library()` the OncinfoUt package.
Then we use its function `caLibraries()` to call all required libraries.
This function will install the missing libraries for you,
among other things that can handle automatically.

The `setExperiment()` function interactively chooses an experiment from your ‘experiments’ folder.
Then we source ‘settings.R’. At the end of this script, we must have a `config` list in the environment.
Let’s see the content of this file:

```
## Load the "merged" experiment config.
config <- loadExperimentConfig(experiment=experiment)

## Enrich config. These are the parts that requires R code
## and cannot be embedded in the yaml files, at least easily.
```

We first call the function `loadExperimentConfig()`, which is in some sense the most important function in the package.
It will load the yaml files and return a list.
Then you see that we add some other stuff to the returning list.
These are the parts that cannot be easily included in the yaml files.
We will get back to this issue later.

The function `chooseFrom()` enables you to interactively choose a subset of experiment tasks.
This step is completely optional. It would be helpful when you want to run just some parts of the whole pipeline.

Now let’s take a look at the basic form of your *application.yaml* file:

```
purpose: "Briefly write down the purpose of this application/pipeline"
log:
  threshold: INFO
proj:
  name: OncinfoUtExamples_DE
  basePath: "~"
resultMidfix: "basic"
params:
  prepare_aml_mds:
    purpose: "Prepare AML and MDS datasets for the next steps"
    taskFolder: aml_mds
    outputFileName: "aml_mds.RData"
  analyzeDE:
    purpose: "Run DE analysis"
    taskFolder: de
  reportDE:
    purpose: "Write the report for the results of the DE analysis."
    taskFolder: de
  plotDE:
    purpose: "Generate the heatmaps for the results of the DE analysis."
    taskFolder: de
datasets:
  Leukemia_2024:
    midPath: Leukemia/2024
```

As you see, we add a `purpose` for the whole application and each task.
This field is mandatory. You will get an error if you do not provide the purposes.
The reason for this behavior is to encourage documentation.

Now let’s see our first experiment yaml file
with the name *2024-08-01\_analyze\_main\_dataset*:

```
purpose: "Run the DE analysis for the combined AML and MDS data"
dataset: Leukemia_2024
tasks:
  - prepare_aml_mds
  - analyzeDE
  - reportDE
params:
  prepare_aml_mds:
    outputType: SYMBOL
  analyzeDE:
    designExpression: "~ label"
  reportDE:
    pvalueCutoff: 0.05
    lfCutoff: 0.2
```

We again write down the purpose of this experiment at the beginning.
Then you can see the list of tasks that must be run for this experiment.
Then in the `params` section, we set the parameters for each one of the tasks.

You can try this example now. Open a fresh R session, and run:

```
setwd(system.file("extdata/examples/proj/OncinfoUtExamples_DE/code/1_basic", package="OncinfoUt"))
source("runall.R")
```

When you run this example, you will notice that there are some files created for you for each task.
One of them is a yaml file starting with “params\_” and the name of the task, then a timestamp.
This is the result of the function `writeParams()` and
is exactly the parameters of the task, saved as a yaml file.
Another one is a RData file which is the result of the function `saveInfo()` in this package.
It writes some info about the R session and system.
We use these to files for future reference
and more confidence on the reproducibility of the results in the future.

### 5.1.1 How does the experiment config look like?

Let’s take a look at the resulting config object, which is a *list* resulting from the function `loadExperimentConfig()`:

```
config <- loadExperimentConfig(experiment="2024-08-01_analyze_main_dataset",
                               experimentsDir=getExampleResource("1_basic/experiments"))
cat(yaml::as.yaml(config))
```

```
## log:
##   threshold: INFO
## proj:
##   name: OncinfoUtExamples_DE
##   basePath: '~'
## resultMidfix: basic
## params:
##   prepare_aml_mds:
##     purpose: Prepare AML and MDS datasets for the next steps
##     taskFolder: aml_mds
##     outputFileName: aml_mds.RData
##     outputType: SYMBOL
##   analyzeDE:
##     purpose: Run DE analysis
##     taskFolder: de
##     designExpression: ~ label
##   reportDE:
##     purpose: Write the report for the results of the DE analysis.
##     taskFolder: de
##     pvalueCutoff: 0.05
##     lfCutoff: 0.2
##   plotDE:
##     purpose: Generate the heatmaps for the results of the DE analysis.
##     taskFolder: de
## datasets:
##   Leukemia_2024:
##     midPath: Leukemia/2024
## purpose: Run the DE analysis for the combined AML and MDS data
## dataset:
##   midPath: Leukemia/2024
##   name: Leukemia_2024
## tasks:
## - prepare_aml_mds
## - analyzeDE
## - reportDE
## experiment: 2024-08-01_analyze_main_dataset
## projResPath: /Users/kazempour/proj/OncinfoUtExamples_DE/result
## experimentPath: /Users/kazempour/proj/OncinfoUtExamples_DE/result/basic/2024-08-01_analyze_main_dataset
## rawPath: /Users/kazempour/proj/OncinfoUtExamples_DE/data/Leukemia/2024
## taskPathS:
## - /Users/kazempour/proj/OncinfoUtExamples_DE/result/basic/2024-08-01_analyze_main_dataset/aml_mds
## - /Users/kazempour/proj/OncinfoUtExamples_DE/result/basic/2024-08-01_analyze_main_dataset/de
## - /Users/kazempour/proj/OncinfoUtExamples_DE/result/basic/2024-08-01_analyze_main_dataset/de
```

As you can see, this list has everything in your application and experiment yaml files.
But there are some more fields in it which we automatically fill for you to use it in your scripts.
One field is `experiment` which is the name of your experiment.
Another one is `experimentPath` which is the main result path for this experiment.
You can use this one in case you want to access other experiment results in yours.
Note how we used `resultMidfix` field in these two paths.
Also note that “~” is expanded here to the actual user folder.
Another one is `dataset`. We changed it a little bit.
In your experiment yaml file, you just mention the name of the dataset to be used.
In the application yaml file, however, you list all `datasets` that you may use in all your experiments.
Each dataset can have its own configuration that you may use throughout your application,
but the field `midPath` is mandatory, though it can be an empty string.

### 5.1.2 How does the task config look like?

The task parameters looks much simpler:

```
config <- loadExperimentConfig(experiment="2024-08-01_analyze_main_dataset",
                               experimentsDir=getExampleResource("1_basic/experiments"))
taskParams <- getTaskParams(config, "reportDE")
```

```
## This task's results will be written in the path: /Users/kazempour/proj/OncinfoUtExamples_DE/result/basic/2024-08-01_analyze_main_dataset/de
```

```
cat(yaml::as.yaml(taskParams))
```

```
## purpose: Write the report for the results of the DE analysis.
## taskFolder: de
## pvalueCutoff: 0.05
## lfCutoff: 0.2
## task: reportDE
## experiment: 2024-08-01_analyze_main_dataset
## rawPath: /Users/kazempour/proj/OncinfoUtExamples_DE/data/Leukemia/2024
## dataset:
##   midPath: Leukemia/2024
##   name: Leukemia_2024
## proj:
##   name: OncinfoUtExamples_DE
##   basePath: '~'
## experimentPath: /Users/kazempour/proj/OncinfoUtExamples_DE/result/basic/2024-08-01_analyze_main_dataset
## taskPathS:
## - /Users/kazempour/proj/OncinfoUtExamples_DE/result/basic/2024-08-01_analyze_main_dataset/aml_mds
## - /Users/kazempour/proj/OncinfoUtExamples_DE/result/basic/2024-08-01_analyze_main_dataset/de
## - /Users/kazempour/proj/OncinfoUtExamples_DE/result/basic/2024-08-01_analyze_main_dataset/de
## taskPath: /Users/kazempour/proj/OncinfoUtExamples_DE/result/basic/2024-08-01_analyze_main_dataset/de
```

Here, you see just the parameters of the specified task.
But still you have access to the important parts of the experiment config outside the `params` field.
A field though is new: `taskPath`. This is the path that this specific task would write its results in.
This path may be shared among different tasks in this experiment if needed.
For this field, we use the value of `taskFolder` which is the name of the task by default,
unless you specify it in one of your config files, most likely the application config file.
Another important new field is `rawPath`. You should put everything regarding your dataset in it.
You may download some dataset and put it there, or better, similar to here,
have some script and task to download or prepare your dataset for the analysis.

## 5.2 Experiments based on each other

Now assume that tomorrow, you want to do another experiment, which is basically the same as the previous one,
but just with different lfc cutoff. You can design this experiment like this:

```
purpose: "Run the DE analysis for the combined AML and MDS data, with high lfc cutoff"
basedOn: 2024-08-01_analyze_main_dataset
params:
  reportDE:
    lfCutoff: 0.3
```

When you are using `basedOn` in your experiment yaml, it will first load that experiment yaml file.
Then will *merge* those yaml files, but getting higher priority to the values that you set in the main one.
So you can override the previous values with new ones.
You can do it on whatever you want. For example, if you want your new experiment to run an additional task,
you can set `tasks` in your new yaml file, and it will replace the previous value in the base experiment.

You can do this recursively and have a chain: EXP2 can be based on EXP1 and EXP3 can be based on EXP2 and so on.
Again we will merge the yaml files for you with higher priority of newer experiments.

Note that although we put the task `prepare_aml_mds` here and in the following experiments,
you do not really need to run it again and again, as it will just overwrite the previously prepared file.
We include it in all experiments for them to be self-contained.
At the same time, we use the function `chooseFrom()` in our `runall.R` script for such circumstances.

## 5.3 Common parameters

Now let’s take a look at the experiment config for `2024-08-02_analyze_main_dataset`:

```
purpose: "Run the DE analysis for the combined AML and MDS data"
dataset: Leukemia_2024
tasks:
  - prepare_aml_mds
  - analyzeDE
  - reportDE
  - plotDE
params:
  common:
    pvalueCutoff: 0.05
    lfCutoff: 0.2
  prepare_aml_mds:
    outputType: SYMBOL
  analyzeDE:
    designExpression: "~ label"
  reportDE:
  plotDE:
```

You can see a `common` section in the parameters. We did this because both `reportDE` and `plotDE` tasks
need the same thresholds for p-value and lfc. We pass anything in this section to all tasks.
Therefore, you will see these parameters even in the `analyzeDE` tasks, although you would not use them.
We simply merge the parameters in the `common` section with the parameters of every task.
If you set the value of the same parameter in both sections of `common` and the task,
the task one would have higher priority and would override the common value.

## 5.4 Run a task on a grid of parameters

We now move to the next step.
To have full working examples, we construct new files.
But you do not need to actually do that in your pipelines.
You may just edit your existing files to extend your pipeline when you want to add new features.

In the previous example, we did an existing experiment on a new value for the parameter `lfCutoff`.
But now assume that we wanted to do it from the start.
We have a handy mechanism to do that, which requires a little adjustment to your scripts and config files.
This is the new experiment config file:

```
purpose: "Run the DE analysis for the combined AML and MDS data"
dataset: Leukemia_2024
tasks:
  - prepare_aml_mds
  - analyzeDE
  - reportDE
params:
  prepare_aml_mds:
    outputType: SYMBOL
  analyzeDE:
    designExpression: "~ label"
  reportDE:
    splitOver:
      - paramName: lfCutoff
        paramVals: [ 0.2 , 0.3 ]
    pvalueCutoff: 0.05
```

The new `runall.R` looks like this:

```
## Load all required libraries throughout all the tasks.
source("libraries.R")

## Interactively choose one of the experiments in your experiment folder.
experiment <- setExperiment()

## Load settings in a list named 'config'.
source("settings.R")

## Use the 'tasks' field in the experiment yaml file.
tasks <- config$tasks
## Optional. Allow the user to interactively choose a subset of tasks.
tasks <- chooseFrom(what="task", allOptions=tasks, doAllowMultiple=TRUE)

## Now loop over all tasks
for (task in tasks[tasks!="none"]) {
    ## Load the "merged" task parameters.
    originalTaskParams <- getTaskParams(config=config, task=task)
    ## Create the output directory of this task
    dir.create(originalTaskParams[['taskPath']], recursive=TRUE, showWarnings=FALSE)
    ## Run the task's code.
    for (sp0 in taskSplitOver(originalTaskParams[['splitOver']])) {
        print(sp0)
        taskParams <- configr::config.list.merge(originalTaskParams, sp0)
        source(file.path("script", paste0(task, ".R")))
    }
}
```

We first use the function `taskSplitOver()` which returns a list.
Then we loop over this list. Every item of this list is a config.
Now we merge this config to our original parameters to have our final task parameters.
Then pass it to the task script.

But you need to have something in mind: your task script must be aware of this splitting.
If you compare the new `reportDE.R` script with the previous one, you will see

```
write.csv(top1, file=file.path(outputPath, paste0("topDE_fc=", lfCutoff,".csv")))
```

instead of simply:

```
write.csv(top1, file=file.path(outputPath, paste0("topDE.csv")))
```

If we had kept the original script, the second run would have overwritten the result of the first run.

You can try this example now. Open a fresh R session, and run:

```
setwd(system.file("extdata/examples/proj/OncinfoUtExamples_DE/code/2_split", package="OncinfoUt"))
source("runall.R")
```

You can generalize this to a config like this:

```
splitOver:
  - paramName: lfCutoff
    paramVals: [ 0.2 , 0.3 ]
  - paramName: pvalueCutoff
    paramVals: [ 0.05, 0.01 ]
```

In this case, you would have 4 runs of the script,
and again you need to change your `reportDE.R` script to respect the value of `pvalueCutoff` in the output file name.

## 5.5 Using glue to generalize your scripts

In this example, we want to use the package `glue`
to replace some parts of the strings with variables and their values or even complicated R codes.

Let’s go a little further in the above example and create more customizable scripts and configs.
We can skip the changes in `reportDE.R` if we generalize it in the first place.
Look at the original script in the example ‘1\_basic’:

```
outputPath <- taskParams[['taskPath']]
pvalueCutoff <- taskParams[['pvalueCutoff']]
lfCutoff <- taskParams[['lfCutoff']]

inputFile <- file.path(taskParams[['taskPathS']]['analyzeDE'], "fitB.RData")

## Getting data:
variableName <- load(inputFile)
fitB <- get(variableName)

## Report:
top1 <- topTable(fitB, number=Inf, p.value=pvalueCutoff, lfc=lfCutoff)
log_info("I found ", nrow(top1), " DE genes with p-value<", pvalueCutoff, " and |lfc|>", lfCutoff)

## Save:
write.csv(top1, file=file.path(outputPath, "topDE.csv"))
```

We change it like this in ‘3\_glue’:

```
inputFile <- taskParams[['inputFile']]
outputFile <- taskParams[['outputFile']]
pvalueCutoff <- taskParams[['pvalueCutoff']]
lfCutoff <- taskParams[['lfCutoff']]

## Getting data:
variableName <- load(inputFile)
fitB <- get(variableName)

## Report:
top1 <- topTable(fitB, number=Inf, p.value=pvalueCutoff, lfc=lfCutoff)
log_info("I found ", nrow(top1), " DE genes with p-value<", pvalueCutoff, " and |lfc|>", lfCutoff)

## Save:
write.csv(top1, file=outputFile)
```

It looks completely flexible now in the input and output, right?
Although the logic of the analysis is completely the same.

We did the same thing for the script `analyzeDE.R`.
Now to use these new flexible scripts, we can use the function `glueStrings()` in the OncinfoUt package.
This function takes some strings and glue them using an environment from a list of parameters.
Let’s take a look at the new experiment config file:

```
purpose: "Run the DE analysis for the combined AML and MDS data"
dataset: Leukemia_2024
tasks:
  - prepare_aml_mds
  - analyzeDE
  - reportDE
params:
  prepare_aml_mds:
    outputType: SYMBOL
  analyzeDE:
    stringsToGlue:
      inputFile: "{rawPath}/aml_mds.RData"
      outputFile: "{taskPath}/fitB.RData"
    designExpression: "~ label"
  reportDE:
    splitOver:
      - paramName: lfCutoff
        paramVals: [ 0.2 , 0.3 ]
      - paramName: pvalueCutoff
        paramVals: [ 0.05 , 0.01 ]
    stringsToGlue:
      inputFile: "{taskPathS['analyzeDE']}/fitB.RData"
      outputFile: "{taskPath}/topDE_fc={lfCutoff}_pval={pvalueCutoff}"
```

and our new `runall.R` script:

```
## Load all required libraries throughout all the tasks.
source("libraries.R")

## Interactively choose one of the experiments in your experiment folder.
experiment <- setExperiment()

## Load settings in a list named 'config'.
source("settings.R")

## Use the 'tasks' field in the experiment yaml file.
tasks <- config$tasks
## Optional. Allow the user to interactively choose a subset of tasks.
tasks <- chooseFrom(what="task", allOptions=tasks, doAllowMultiple=TRUE)

## Now loop over all tasks
for (task in tasks[tasks!="none"]) {
    ## Load the "merged" task parameters.
    originalTaskParams <- getTaskParams(config=config, task=task)
    ## Create the output directory of this task
    dir.create(originalTaskParams[['taskPath']], recursive=TRUE, showWarnings=FALSE)
    ## Run the task's code.
    for (sp0 in taskSplitOver(originalTaskParams[['splitOver']])) {
        print(sp0)
        taskParams <- configr::config.list.merge(originalTaskParams, sp0)
        if ('stringsToGlue' %in% names(taskParams)) {
            glueParams <- glueStrings(templates=taskParams[['stringsToGlue']], params=taskParams)
            taskParams <- configr::config.list.merge(taskParams, glueParams)
        }
        source(file.path("script", paste0(task, ".R")))
    }
}
```

What has happened here? In fact, we moved the *logics* in the naming of input and output files
outside our script to our config files to make the scripts more flexible and reusable.
Note how the output of `analyzeDE` task is connected to the input of the task `reportDE`.

Note that in most cases, it is even better to put this kind of `stringsToGlue` field
even in your application yaml file, as this logic is usually shared among your experiments.
Of course, you can override the application `stringsToGlue` with your experiment’s as usual, if needed.

You can try this example now. Open a fresh R session, and run:

```
setwd(system.file("extdata/examples/proj/OncinfoUtExamples_DE/code/3_glue", package="OncinfoUt"))
source("runall.R")
```

## 5.6 Using a SLURM cluster

Now assume that you have a task that cannot be run on your laptop and requires lots of resources.
So we would need to submit a job in a SLURM cluster, TACC for example.
In such cases, we have a helper function `writeJobScripts()` to help you create a job file
using your config files to be submitted on the cluster.
Let’s do our previous DE analysis, but this time in the SLURM cluster.
We do this here just for the analyzeDE task, as it must be the most resource consuming one.

The task script which is called from `runall.R` is now literally one line of code:

When you source the `runall.R` script, it will just call `writeJobScripts()` function.
This function will write to script files for you. We discuss them in more details later.

The new application config is this:

```
purpose: "Briefly write down the purpose of this application/pipeline"
log:
  threshold: INFO
proj:
  name: OncinfoUtExamples_DE
  basePath: "~"
resultMidfix: "SLURM"
params:
  analyzeDE:
    purpose: "Run DE analysis on a SLURM cluster"
    taskFolder: de
    slurm:
      name: deAn
      shell: |
        APPLICATION_PATH=`system.file("extdata/examples/proj/OncinfoUtExamples_DE/code/4_slurm", package="OncinfoUt")`
        Rscript --vanilla "$APPLICATION_PATH"/script/helper/analyzeDE.R "$APPLICATION_PATH"/experiments `experiment`
datasets:
  Leukemia_2024:
    midPath: Leukemia/2024
```

and the new experiment config is this:

```
purpose: "Run the DE analysis for the combined AML and MDS data"
dataset: Leukemia_2024
proj:
  basePath: "~"  ## Change this to the actual path that you expect to have your data and results on the cluster
tasks:
  - analyzeDE
params:
  analyzeDE:
    stringsToGlue:
      inputFile: "{rawPath}/aml_mds.RData"
      outputFile: "{taskPath}/fitB.RData"
    designExpression: "~ label"
    slurm:
      emailAddress: someone@uthscsa.edu
      execTime: "01:00:00"
      queue: development
```

What we add in both is a section `slurm`.
As usual, we put everything that is shared among experiments in the application config.
Here, the name of the job files and the shell script that must be run for this task.
The experiment config consists of things like the execution time requested from the cluster,
the queue you want to submit the job in, your email address, etc.
For the full set of options, please go to the documentations.

Now run the example in your local machine first:

```
setwd(system.file("extdata/examples/proj/OncinfoUtExamples_DE/code/4_slurm", package="OncinfoUt"))
source("runall.R")
```

As we pointed out, you must see two files written in your task result folder inside a folder named *job*.
The name of both files are “deAn” (what we put in our config).
One of the written files has the extension `.slurm` and must be submitted in the cluster with `sbatch` command.
This file contains some information for the `sbatch` command and as you may see,
we can configure things like the email address or queue in our configuration files.
At the end, it calls the other written file with the extension `.sh`.
In this file, you can see the code that we put in our application yaml file,
but with the things between *ticks* expanded as R expressions.
For example, `` `experiment` `` has expanded to the actual value of experiment.
What happens behind the curtains is that we use the `glue` package again,
but this time using *ticks* for the opening and ending of expressions.

To actually run the task in the cluster,
you need to source the `runall.R` script in the login node of the cluster first.
Also, usually the home folder is not a good option to put the data and results.
Therefore, you need to change your `proj.basePath` to something like a work folder.
Then you will have your `job` folder in the cluster and can run `sbatch` on the `.slurm` file.

If you inspect the actual script that would be run at the end, i.e. `helper/analyzeDE.R`,
you will find out that we have changed it a little bit.
The reason is that this script would not be sourced from our `runall.R` script anymore.
So we cannot directly use `taskParams` in it.
You can use two designs here. One design that we used here is that
you can pass the experiment name and your experiments folder to it.
Now the script knows these two required things to start with the `loadExperimentConfig()` function.
Then it can create the `taskParams` object itself and use it.
We must mention that here, it is a good practice to keep your original script intact,
and write a wrapper around it to do this config loading. We wanted to keep our example simple.
The main advantage of this design is that it is completely flexible and simple.
Another design would be to write your script in such a way that accepts its parameters fully from the command line.
Then in your `slurm.shell` section, you may use your existing task parameters
and pass them one by one as required to your script.
The advantage of this design is that you will have a very flexible script which can be used anywhere.
In fact if you want to use existing tools and scrips that accepts their parameter through the command line,
you have to use this design. For example, something like this,
assuming you have `quality`, `inputFile`, and `outputFile` in your task parameters:

```
slurm:
  shell: |
    samtools view -b "`inputFile`" -q `quality`> "`outputFile`"
```

The function `writeJobScripts()` has access to all of your task parameters
and can glue those parameters for you in the resulting scripts.
Just be aware of the characters that must be escaped in shell.
For example, we have put `` `inputFile` `` inside quotations
which will handle the cases where the input file name consists of spaces for example.

## 5.7 Some notes

There are some advanced configurations that can be done
with splitting and gluing and depending on other experiments.
For example, you can split not only on simple parameters like numbers,
but also on some complex configurations.
We have used such patterns in some of our applications.

The function `glueStrings()` is limited to simple string parameters.
It is still completely sufficient for many applications.
But if for any reason, you need some complex configurations
that can not be handled by the available features in this package,
feel free to add some lines of codes in your `settings.R` script or your task scripts.
We do not recommend it, as it will couple your scripts with some configuration logics,
but sometimes it is not easily avoidable anyway.
